# Health policy and controlling Covid-19 in England: sociological insights

**DOI:** 10.35241/emeraldopenres.13726.2

**Published:** 2020-07-29

**Authors:** Michael Calnan

**Affiliations:** 1 School of Social Policy, Sociology and Social Research, University of Kent, Canterbury, Kent, CT27NF, UK

**Keywords:** Covid-19, Health Policy, Power, Government; NHS, England

## Abstract

The global Covid-19 pandemic is posing considerable challenges for governments throughout the world and has and will have a significant influence on the shape of peoples social and economic life and wellbeing in the short and longer term. This opinion paper discusses the current health policy response adopted in England to control or manage the epidemic and identifies the key sociological and political influences which have shaped these policies. Drawing on the theoretical approach set out in his recent book, which emphasises the interplay of ****powerful structural and economic interest groups, the author will consider the influence of the key players. Government policy has tied itself to scientific and medical evidence and protecting the NHS so the key roles of the medical profession, public health scientific community and NHS management and their respective and relative powerful influences will be discussed. The government needs the support of the public if their policies are to be successful, so how have the government addressed maintaining public trust in this ‘crisis’ and how much trust do the public have in the government and what has influenced it? The strong emphasis on social distancing and social isolation in the national government policy response to Covid-19 has placed an increasing public reliance on the traditional and social media for sources of information so how the media has framed the policy will be considered. One policy aim is for an effective vaccine and the influence of the drug industry in its development is discussed. Finally, the role of the state will be discussed and what has shaped its social and economic policies.

## Background

The death toll in England from Covid-19 has claimed to have peaked on April 8 ^th^ 2020 ( Dorling, 2020) or least reached its the first peak, and the infection rate, at least in the community, appears to be declining. The overall number of Covid-19 related deaths reported by July 22nd 2020 was 45,422 in the UK and 40,828 in England although both might be an underestimate as at 30/06/2020 the excess mortality for Covid-19 related deaths in the UK is estimated at 64,451. Men are more likely to die from the virus than women and the risk of dying from Covid-19 is age related with older people most at risk. The overall aim of this opinion paper is to critically examine national government policy in England which, over the last five months or so, has attempted to control or manage the Covid-19 pandemic. The paper will begin with a descriptive account characterising the policy to date and then provide a critical analysis of why it has taken the shape that it has identifying the key influences and interplay of powerful interest groups.

## The policy response in England

The government policy response might be described to date as one which was characterised as reactive ( McConalogue & Knox, 2020) was slower to develop than in other countries, which significantly changed its direction at least earlier on, and lacked a clear and consistent communication strategy particularly about exit plans. It has also been tied to medical science, or one brand of it ( Paton, 2020) although this has loosened in relation to the more recent policies involving the relaxation of the stringent control measures.

The government response to Covid-19 in England was originally described in terms of three phases which were: containment (e.g. contact tracing, education for effective hand washing), delay (which aims to flatten the peak of the outbreak to protect the NHS and provide time for research to develop effective tests, treatments and vaccines) and mitigation (based on the idea of 'herd immunity', where the epidemic should be allowed to run its course to allow the population to build up resistance to it. Mitigation would be introduced to limit the number of deaths through protecting the most vulnerable, but the government would not need to totally eradicate the disease). The general approach was presented as evidence-based, with the Scientific Advisory Group for Emergencies (SAGE) appearing to actively provide advice and some argue, drive policy ( McConalogue & Knox, 2020). An emphasis was placed on the timing of the introduction of policies to maximise their effectiveness based on the scientific evidence ( Calnan, 2020a; WHO, 2020).

Containment was the initial policy, but on March 12 ^th^ the government announced it was moving from the containment to the delay phase with the caveat that this policy would not be as ‘draconian’ as adopted in other countries. The Prime Minister (PM) raised his profile and, in the first of the televised daily media briefings/updates, was flanked by two medical and scientific experts. The PM gave the ‘honest ‘message that people might lose their loved ones suggesting it should be taken seriously as it was the ‘worst public health crisis for a generation’ ( Paton, 2020; The Health Foundation, 2020).

This painful message was difficult for the public to accept and their response in combination with new scientific evidence appears to have led, a few days later, to a shift in policy. This epidemiological modelling evidence ( Ferguson *et al*., 2020) was based on the experience mainly from China. The report recommended suppression as the policy option – apparently not an option seriously considered initially by the modellers as it was not expected to be acceptable both political and socially. The policy of suppression aims to reverse epidemic growth rather than mitigation which according to the report would have led to the overwhelming of the health system and the loss of hundreds of thousands of lives. Thus, the government moved from its delay strategy towards a policy of suppression of the transmission. Schools were to remain open, but the government advised the public to adopt social distancing and curtail social activities such as the use of pubs/restaurants, to work from home where possible, with individual/household isolation for 7 or 14 days for those with symptoms. This policy put an emphasis on advising the public rather than instructing them as adopted in other countries and seems to reflect the influence of behavioural psychological expertise, or some strands of it, with concern expressed by some government representatives about public behavioural fatigue ( Oliver, 2020).

The government then began to take more stringent measures such as increasing control and limitations over foreign travel, shutdown of schools in England and banning the gatherings of more than two people (excluding people who live together). 27 million households were to be sent letters highlighting the need to continue social distancing and to only leave the house for shopping for basic necessities, exercise, any medical need and travelling to work if unable to work from home. There was a change in the tone of the message from government as the emphasis shifted to ‘instructing’ rather than ‘advising’ the public. Emergency legal powers were introduced to enforce these measures suggesting that the public, or at least some sections of the public, could not be trusted to behave responsibly. The police have been given the power to fine people(which have been increased in the most recent review) who are not adhering to these measures and concern was then expressed about a lack of consistency in the exercise of these powers by the police. There was also the potential threat to civil rights if there is a further extension of such coercive policies although these powers were required to be renewed every six months. Survey evidence suggests the public were unaware of the strength of many of these powers available to the police ( Duffy, 2020). The extent to which the public did not adhere to the restrictions is difficult to judge, although overall adherence continued to be relatively high ( Duffy, 2020).

Policies also focused on trying to free up and repurpose capacity within the hospital acute sector. The NHS negotiated block contracts with private hospitals who have a relatively small number of intensive care beds but would treat non-urgent NHS patients. The policy discourse has emphasised a partnership between the two sectors ( West, 2020) particularly in time of crisis although others have seen it a further sign of creeping privatisation encouraged by the government:

‘ *Deloitte, KPMG, Serco, Sodexo, Mitie, Boots and the US data mining group Palantir have secured taxpayer-funded commissions to manage Covid-19 drive-in testing centres, the purchasing of personal protective equipment (PPE) and the building of Nightingale hospitals.’* ( Garside & Neate, 2020).

This apparent need to turn to the private sector for national emergencies has been explained by the lack of investment in the infrastructure of the NHS and privatisation over the last ten years ( Lawrence *et al*., 2020).

The building of temporary Nightingale hospitals throughout the country (one in East London had a full capacity of 4,000 beds, although it has recently been mothballed as underused but may be required if there is a second peak) has expanded the number of intensive care beds and recruiting retired clinicians has begun to address the shortfall in staff with 500 trained staff being reported as being recruited from that source. The government said it would write off £13.4 billion of historic NHS debt ( The Health Foundation, 2020) so that hospital trusts are in a "stronger position" to deal with the outbreak. Policies aimed at increasing the supply and distribution of protective equipment for frontline staff such as gowns and the availability of ventilators and the expansion of testing of staff and patients/public have proved more problematic to implement and have been a focus of widespread criticism ( Calnan, 2020a).

The recognition of the need for systematic testing, although abandoned initially, led the Minister for Health to set an ‘audacious’ target that across five different pillars 100,000 tests would be carried out per day by the end of April, while during Prime Minister’s Questions the next stated target was 200,000 a day by the end of the month ( Buck, 2020). In the weeks leading up to this deadline, the level of testing for the virus in staff and patients continued to be significantly below that target but by April 30 ^th^ the number of tests reported to have been carried out reached well over that figure (122,347) although it is suggested that this figure is artificially inflated by the inclusion of self-testing kits which have been posted to be people homes but not yet completed. This daily level of testing has varied since then not consistently hitting the targets and it is claimed at May 31 ^st^, 2020 that the capacity for testing is over 200,000 per day, with number actually being tested well under that (115,000).

The government also has renewed community contact tracing alongside a mobile app in mid-May, mobilising 18,000 contact tracers including 3,000 health professionals. This is being run nationally by a private outsourced company with limited involvement of local public health expertise and experience in this type of work. This was trialled in the Isle of Wight with mixed results and although contact tracing has begun, it appears that it will not now be fully operational using a new app until later in the year . The use of this system has raised questions about threats to privacy and security and concerns about victimisation of those seen to be spreaders of the virus.

There is some still uncertainty about how far overall this is a coherent, phased, testing strategy ( Godlee, 2020a). For example, as Grassly *et al*. (2020) concluded:

*‘Testing is essential for pandemic surveillance but its direct contribution to the prevention of transmission is likely to be limited to patients, HCWs and other high-risk groups’.*

The restrictions associated with the governments suppression of transmission policy were reviewed and the cabinet agreed to a further extension for three weeks. There was increasing pressure for the government to be transparent and outline its exit plans to the public and it set out five tests before easing of the restrictions can take place which were: Making sure the NHS could cope; A "sustained and consistent" fall in the daily death rate; reliable data showing the rate of infection was decreasing to "manageable levels"; ensuring the supply of tests and Personal Protective Equipment (PPE) could meet future demand and being confident any adjustments would not risk a second peak. It is suggested that relaxation of social distancing measures will depend on the reproductive rate (R) being and staying below one. This R rate has informed the three-step conditional plan for gradually easing the restrictions with a non- specific timetable although social distancing rules are still in place. For example, step one recommended that from May 13th that people who "can't work from home" are "actively encouraged to go to work" but to avoid public transport if they can; step two includes phased reopening of primary schools in June at the earliest and step three the reopening of public places in July. The government also changed its public message to "stay alert, control the virus, save lives" from "stay at home”. A Covid-19 alert system was introduced by the government to track the virus which ranks the threat level on a scale of one-five with England currently still at stage four despite the easing of restrictions.. A change in policy is reflected in the recommendation of use of face masks by the public when in confined public spaces and it is now compulsory on public transport and in hospitals and in shops and supermarkets ( Calnan, 2020a). These policies slightly differed between the devolved countries in the UK and this variation in policy continues to be evident ( Brown, 2020).

 Policies over the last three months have focused on further easing of the stringent restrictions which were put in place in March 2020.These policies or the’ road map’, have been in the main tied, although not always, to the five tests for the easing of the restrictions which were set out previously and were to be linked to the Covid 19 system of alert levels which was downgraded from 4 to 3 on 19/06/2020. There have been a number of policies introduced gradually over this period but perhaps the most significant were the reduction in the social distancing rule from 2 to I metre plus; the introduction and then revision of quarantine laws for travellers from selected countries to this country; the compulsory wearing of face coverings on public transport, in hospitals and more recently in shops; the re-opening of non -essential shops and the hospitality and some parts of the sports/entertainment industry; the permission for two households of any size were able to meet indoors and outside and for the vulnerable no longer needing to shield from August ( The Health foundation, 2020). One policy, which has been the focus of a vociferous debate, was the plan for all primary school years to go back to school before the end of the summer term but this was not implemented by the government as it was inoperable. Schools will not now fully open until September.

Achieving each of the five tests has also proved problematic not least ensuring the supply of tests. There was considerable political rhetoric about the potential of the test and trace system but the bespoke mobile app was dropped and there have been communication problems between the centralised system being run by an outsourced company and the local public health agencies who have considerable experience in this field. It has been reported that £10bn has been spent on this system and £15bn on PPE for frontline staff which are also provided by the private sector which it is argued suggest evidence of governments continued support for privatisation of the health service ( Iacobucci, 2020a). One of the five tests also included the need not to risk a second peak but there has been evidence of second peaks in some local areas such as Leicester where stringent restrictive measures have been reintroduced. This outbreak has been claimed to be linked to the risks associated with the so called’ sweat shops’ of the garment industry found in this locality ( Bland, 2020). Once again poor relationships between national and local public health agencies are claimed to be a reason for the slow response to this outbreak as after initialling denying access it was agreed to provide local authorities with the data required for test and trace ( Torjeson, 2020). However, the government has now given local authorities ‘lightning’ lock down powers to close shops etc. Medical scientists have argued for the need to be prepared for a second wave of Covid-19 in the coming winter with a prediction of further significant loss of life ( Iacobucci, 2020b). The government has subsequently pledged an extra £3bn to ease winter pressures on the NHS in England. The government has also set up a new Joint Biosecurity Centre(JBC) ( Vize, 2020) which aims to play a lead role in coordinating the response to Covid-19 and complement the work of SAGE which will focus on longer term problems. However, it has been suggested ( Vize, 2020) that in the future the JBC might take over the role of Public Health England in relation to monitoring infectious diseases because:

*‘Public Health England will be lined up by the government for a lot of the blame, perhaps partly on the grounds that it lost its focus on infectious diseases in favour of lifestyle illnesses. If Public Health England does not survive, it is possible to envisage the Joint Biosecurity Centre ending up as a permanent fixture with some of PHE’s communicable disease functions’ ( Vize, 2020 p2)*

The government addressed the economic consequences of their policies with an emphasis in policy on protecting the health of both the public and the economy and they have been attempting to juggle these priorities throughout. The initial response was in the spring budget which was followed by a much stronger package of measures introduced to provide support for businesses, supporting wages of vulnerable staff, addressing workers’ rights and pay during the ‘crisis’ and support for the self-employed. There has also been a commitment to charitable organisations who provide frontline care for older and vulnerable people ( Calnan, 2020a). Some of these schemes, such as the furloughing policy, have been extended until October when it will finish raising concerns about high levels of unemployment. This has involved significant public expenditure and intervention in the market economy although the treasury describes many of these measures as temporary and unsustainable. More recently with the increasing emphasis on resuscitating the economy a plethora of policies have been introduced by the Chancellor of the Exchequer including a Jobs Retention Bonus, giving £1,000 to businesses who bring back employees from furlough; a temporary VAT cut for hospitality and tourism - down from 20% to 5% ;An "Eat Out to Help Out" scheme for August, giving a discount to people eating at cafes, restaurants and pubs ; a rise in the threshold of stamp duty from £125,000 to £500,000 ;A "kickstart scheme" to get unemployed 16 to 24-year-old into work and new payments for businesses hiring apprentices ( The Health Foundation, 2020)

## Explaining the shape of the policy response

The shape of health policy making in England has been shown to be influenced by the interplay of powerful structural and economic interests ( Calnan, 2020b). The ‘loose’ theoretical approach adopted here combines a number of theoretical perspectives ie structural pluralism and political economy. It is essentially a socio-political perspective on health policy making and the policy arena of health and health care contains a number of powerful interest groups. The policy response to Covid-19 clearly reflects this and a range of players have been and are involved which includes the government and its representatives, NHS managers, scientific advisers and the scientific community, the medical profession, their representatives and clinical practitioners, the allied professions including nursing practitioners and social care agencies and workers, the private health and social care sector, the commercial sector who produce vaccines, ventilators and PPE, the media in the public and private sectors and the public, patients and their informal carers.

## The influence of the medical profession

The focus here will be on the key players and one of these, as with other areas of health policy making in England ( Calnan, 2020b), has been the medical profession and medical and public health expert advisers have been particularly prominent in shaping this policy response through advice. Government ministers have consistently invoked that decisions will depend on ‘the science’. However, the medical profession has been divided in their support of government policy with government medical officers supporting, but those who are more independent of it criticising the overall approach and those working in the NHS suggesting that services were inadequate particularly vociferous in relation to protecting clinical staff on the ‘frontline’. The emphasis in one sociological narrative of medical professionalism is that clinicians are driven by altruistic values ( Calnan, 2020b) and this seems to be supported by the argument that it is primarily the professional commitment of the staff with its public sector and ethical values which have sustained the NHS rather than government support:

*“the NHS relies on the heroic professionalism and planning skills of its staff, and the self-control of the public”*. ( Abbasi, 2020a, P1).

One particular focus of criticism has been on the demise of public health in England particularly in its role in testing and contact tracing:

*‘Too little, too late, too flawed…How did a country with an international reputation for public health get it so wrong? Their answer is a sad litany of past and present decisions that have fragmented, decimated, and marginalised public health in the run up to this moment when it is most needed. Their overriding message is that clear leadership from the centre needs to be matched with strong operational capacity at the local level. The UK currently has neither’* ( Godlee, 2020b, P1).

## The influence of scientific medical expertise

Evidence-based policy, or at least the discourse about ‘what works counts’, was popular with the Labour administration in the early part of this century ( Calnan, 2020b). More recently, however, there seems to have been what might be described as a second ‘cultural turn’ which is reflected in the significant populist social and political upheaval in the Western world and the emergence of the post-truth society with a loss of trust in experts. Thus, this emphasis in the governments’ response to Covid-19 on trust in scientific experts appeared to reflect a marked shift in approach from a populist government, where some senior members had famously explicitly articulated a lack of faith in experts. It suggests a ‘rational’ approach to policy making although it is difficult to judge precisely how far the evidence influenced decision making. Studies ( Cameron *et al*., 2011) have suggested that evidence is sometimes used by policy makers as ammunition to justify policies that are favoured politically. Yet the media daily news briefings consistently involved both politician and medical/public health scientists and the foreign secretary (07/04/2020) referred to an evidence-based approach to shaping policies. This linear or rational approach has also been challenged for treating the evidence base, such as the mathematical models ( Ferguson *et al*., 2020), which led to the shift in government policy, as rigid and as ‘boundary objects’ ( Rhodes & Lancaster, 2020) when they should be seen as more fluid and the social process of the development of the evidence base as more emergent and adaptive. One particular problem in this context is the lack of evidence and uncertainties about the transmission of the virus and its control. The government appears to have deferred to science, or one brand of science ( Paton, 2020), to guide their policy although it is argued that this depoliticising strategy also could be used as a ploy for shifting responsibility from what is essentially political decision making about policy ( McConalogue & Knox, 2020). However, despite scientific advice becoming front stage with the subsequent visibility of its uncertainties and limitations there has been some but limited criticism of this advice, at least from citizens. The government stance appears to be supported by the public as survey evidence suggests that the public have trust in science and research ( Open Knowledge Foundation, 2020) but prefer data to be openly available for checking and they oppose restricting the public’s right to information. More recently, the government has made more explicit that the role of medical scientists is to provide advice and the government to make political decisions so the level of scientific support for recent government policy of lifting the restrictions is difficult to judge

Scientific opinion, however, has been divided, and there was a call for SAGE to publish its evidence and be more transparent which it eventually has ( Government Office for Science, 2020). Doubts were also raised about how independent the SAGE committee is in terms of its advice and recommendations because of conflicts of interests e.g. advisers employed by the government although even they are reported to have been in open revolt over the Cummings controversy ( Abbasi, 2020b). There also is a suggestion that the scientists with the appropriate expertise were not consulted, for example in relation to testing ( Buck, 2020), implying that some scientific disciplines had a more powerful influence on policy than others.

## The influence of NHS managers

NHS managers have also been key players as within the confines of their budgets they are expected to implement government policy although they may have more discretion and latitude in their decision making than the government would like( Campbell, 2020). One of the stated aims of the suppression policy was to protect the NHS from being overwhelmed. There are conflicting narratives about the position of the NHS in relation to its ability to have managed an epidemic of infectious disease. There is evidence which suggests that the NHS appears to be as prepared as any other health care system to meet the challenges of the demands of this unexpected pandemic. For example, in 2019 on a GHS health care security measuring preparedness the United Kingdom came second out of 195 countries with an index score of 77.9 out of 100. However, the report concluded that national health security is fundamentally weak around the world, that no country is fully prepared for epidemics or pandemics, and that every country has important gaps to address ( GHS, 2019). In contrast, there are other reports ( NAO, 2020) that the recommendations for epidemic preparedness were not prioritised or implemented due to lack of investment. For example, a contingency planning exercise conducted in October 2016 entitled *Exercise Cygnus*, involving national, regional and local government bodies, highlighted, amongst other things, the lack of PPE stocks particularly gowns but their recommendations were ignored and not published, although there is now a campaign to make the reports’ recommendations public ( Dyer, 2020). Little is known about the exercise – or the confidential recommendations that followed from it – other than it confirmed significant gaps in the country’s preparedness.

Other evidence suggests that the NHS is not performing well and has been performing at the limits of its capacity for some time, which probably reflects the chronic lack of investment over the last decade ( Calnan, 2020b). For example, it is argued that Hospital Accident and Emergency (A&E) services ( Cook, 2020) are a good barometer of the state of NHS performance; in the last few years A&E services have consistently failed to meet four-hour targets. This raises questions about its resilience when under extreme pressure and as the WHO recently stated:

“ *Following chronic underfunding and a period of austerity, general acute hospital bed capacity has fallen in the last 20 years in the UK. Prior to the COVID-19 pandemic, hospitals frequently ran at 92% occupancy and often over 95% occupancy in winter, which is well over the capacity deemed to be safe”.* ( https://www.kingsfund.org.uk/publications/nhs-hospital-bed-numbers).

The eventual emphasis on more stringent suppression policy measures appears to be justified by the evidence but there has been much criticism, particularly from independent clinical and public health scientists, of the slower response of the government and NHS management to the outbreak and specifically their inability to learn from the experience of other countries:

*“The UK Government’s Contain–Delay–Mitigate–Research strategy failed. It failed, in part, because ministers didn’t follow WHO’s advice to “test, test, test” every suspected case. They didn’t isolate and quarantine. They didn’t contact trace. These basic principles of public health and infectious disease control were ignored, for reasons that remain opaque. The UK now has a new plan—Suppress–Shield–Treat–Palliate. But this plan, agreed far too late in the course of the outbreak, has left the NHS wholly unprepared for the surge of severely and critically ill patients that will soon come.”* ( Horton, 2020).

This was a view shared by the majority of the public which, according to survey evidence, showed that 56% agreed that government measures were taken too late ( Ipsos Mori, 2020). Germany and South Korea are countries which were seen to be successful in the reduction of their infection and mortality rate from Covid-19. For example, Germany’s policy approach was seen to be consistent and clear from the outset with an emphasis on testing as the following quotation suggests:

*“Berlin’s strategy has nevertheless held up an unforgiving mirror to Britain’s government. This is not just with regard to NHS capacity — tuned more for resource efficiency than resilience — but the quality and pace of decision making. The charge: that Britain’s strategy twisted and turned, squandering precious time. “It just wasn't consistent. They tested various strategies and rejected them,” said Martin Stuermer, a virologist at IMD Labor in Frankfurt. “They had this plan to allow life to go on but ensure that elderly people were protected. But then they abandoned that. And they weren't prepared for mass testing. but the main problem was that the government just didn't chart a clear course in this crisis — unlike the German government*. *”* ( Barker *et al*., 2020).

South Korea’s approach to testing, which involved devolution to the regions and drawing on local testing infrastructures, was not adopted in England, where a centralised system was favoured sequentially expanding outwards using public service laboratories although this was halted primarily due to limited capacity ( McCurry, 2020).

The prioritisation of hospital care for Covid 19 patients also led to a decline in referrals for cancer surgery, in young people seeking help for mental health distress and the use of A&E and primary care services. This might have freed up hospital capacity but suggests a build-up of untreated physical and mental health problems, which has been described as the ‘parallel epidemic’. Certainly, the social isolation policies may have not only increased loneliness and/or exacerbated mental health distress ( Williams *et al*., 2020) but also has led to significant rise in calls for help for domestic abuse ( Duffy, 2020).

## The influence of social care providers

The focus in government policy had been on hospital care but the pandemic has had major consequences for both primary and social care and other public services which were not until recently part of this policy conversation. Social care providers are responsible for caring for the most vulnerable, and care homes are settings which, like hospitals, are currently where there is one of the greatest risks of transmission of the virus. The recognition of infections and deaths from the virus in social care settings remained invisible in the reporting of official statistics, at least in the early stages, and some of the deaths of patients with dementia may not have had a diagnosis of Covid-19 on their death certificate ( Booth, 2020). Social care workers and their clients did not appear to be a priority for testing and for PPE. The limited service has suffered from cuts in funding, as have the third-sector organisations, who in some areas are the primary providers of social care. The importance of social care provision may have belatedly been recognised as its workers have now been given a distinctive ‘brand’ to match NHS staff. This crisis has highlighted the difference, and lack of integration ( NAO, 2020), between the NHS and social care in terms of funding and priorities with the devolved and fragmented nature of social care based mainly in the private sector and the low status of its workers , which stands in marked contrast to the publicly funded and centrally organised NHS with a workforce containing high status professionals ( Sloggett, 2020). It also raises questions about the extent of financial responsibility that the government and local authorities have or should have for care and protection in this sector, e.g. providing PPE.

## The role of the public

The public also had a number of key roles to play through adhering to government policy and though collective action. The PM emphasised the salience of public trust for enhancing adherence to government policies. There are a number of policies which are used to build or maintain trust ( Bachman *et al.*, 2015), but perhaps a more common policy, is focusing on enhancing accountability and transparency; the daily media Covid-19 briefings might have been seen as a strategy for building public trust through enhancing transparency about the risks and uncertainties as it displays honesty and integrity, i.e. being seen ‘to level with people’. This raises the question of how uncertainties should be managed and if they should be made explicit thereby making decisions more transparent, accountable and democratic or ignored and be bracketed off ( Calnan, 2020b). However, transparency poses problems for governments particularly in contexts of heightened uncertainties, such as with this pandemic, as greater transparency may enhance trust in that governments may appear to working in the public interest but on the other hand it might increase exposure to lack of confidence in policy decisions and its implementation. Thus, for example, shifts in policy or poor communication giving unclear, confusing and contradictory messages, e.g. about the reasons for the lack of availability of PPE, about the changes in the message to the public or the recommendations to use face masks, can undermine confidence in competence. Certainly, there is evidence that the disclosure of uncertainties has only a small, negative impact on public trust ( Van der Bles *et al.,* 2020) and thus provides support for a policy of increased transparency. 

Evidence from surveys of the public showed majority support for government policy in the early days as it was evident that the stringent policies were necessary given the media images of other countries such as Italy and reports of ‘brutal’ rationing decisions about gaining access to ventilators. However, more recently, public support for government policy has begun to wane, not least in the light of media criticism about the lack of leadership and problems with the provision of PPE for frontline staff, expanding testing for essential workers and patients, the recent controversy about the governments senior advisor, Dominic Cummings, breaking the rules associated with the lockdown and the problems implementing the track and trace system. Thus, trust in the UK government as a source of information about coronavirus has declined substantially since April. 48% rated the government relatively trustworthy in late May, down from 67% six weeks earlier ( Fletcher *et al*., 2020). More recent survey evidence confirms this trend ( Opinium, 2020) with 47% not approving of government policies compared with 34% who do and this decline in approval is mainly related to the public feeling that the government are underreacting.

Collective social action and social trust is also being called for in these policies, which involves the public trusting in one another to social distance and having some responsibility for the vulnerable and older people - a form of social and altruistic trust ( Calnan *et al.*, 2020). The success of this will depend on societal solidarity and Brexit has led to or exacerbated social divisions and a lack of societal cohesion although it has been manifested more positively in the recruitment of volunteers (750,000) to help the NHS and the weekly public applause for NHS staff. However, this might reflect trust or loyalty to the institution of the NHS which has its own social capital rather than trust in government policy. It is argued that health care systems are embedded in institutional contexts ( Blank & Burau, 2014) and do not just produce healthcare to improve health but they can establish the social norms that shape human action and therefore act as a repository and producer of wider social value ( Gilson, 2006). These norms can help establish a moral community whom you can trust, and they may provide the basis for generalized trust. Thus, while they may have been some ambivalence in public attitudes towards government policy trust was enhanced by public support for the NHS as an institution ( Godlee, 2020c), which also reflected nationalistic values ( Fitzgerald *et al*., 2020).

The extent to which the government feels that they can have trust in the public’s actions may have shaped their decisions about relaxation of the restrictions. The emphasis in recent government policy has been on shifting the responsibility towards individuals (and employers) to make ‘responsible risk judgements’ such as in relation to decisions to return to work and on the more ‘moral’ message of following the publics civic duty of adhering to self isolation in relation to test and trace. In some countries such as Sweden ( Tragarah & Ozkrmh, 2020) there is evidence of mutual trust between the public and the government, which might have suggested that stringent state control measures were not seen to be necessary although the effectiveness of such policies is now in some doubt given the relatively high death rate at least compared with other Scandinavian countries.

One key characteristic of recent health service policy in England has been the focus on public and patient involvement in health care and the importance placed on shared decision making and patient choice ( Calnan, 2020b). The mantra associated with the reforms in 2012 Health and Social Care Act was ‘ *no decision about me without me’* which was derived from the disabled peoples movement. Evidence suggests ( ONS, 2020a) people with disabilities are more at risk of dying from Covid 19 than those without disabilities and such a pattern was more marked among the younger age groups than the older age groups. However, the voice and experience of the patient and their informal carers seems, at least at present, to have had little prominence in the policy conversation although the consultation relationship between clinician and patient has been changed as a result of digitilisation which has been accelerated by the onset of the pandemic and social distancing. 

## The influence of the pharmaceutical industry

In the early stages of government policy, one of the aims of the delaying of the transmission of the virus was to create the space for science to develop an effective vaccination and/or treatment. This involves a key role for the pharmaceutical industry which is in the private sector in England and where much of the research and development for new drugs and vaccines is carried out ( Calnan, 2020b). There is some, albeit cautious, optimism about both and having hope, as well as trust, has been shown as a means for bridging or managing uncertainty which has characterised many aspects of this pandemic ( Brown & Calnan, 2012). Effective drugs may be on the market before a suitable vaccine ( Mullard, 2020a), although there is intense competition. Numerous candidates are being trialled such as those used in the treatment or management of Ebola, Remdesivir, which has recently been authorised by the US Food and Drug Administration for use in emergencies and also endorsed in Australia although recent evidence ( Day, 2020) has raised doubts about its effectiveness. Dexamethasone which is more widely used for the treatment of other diseases is a less expensive option. However, government policy has provided more financial support for a vaccine being developed in the UK which might be seen not only as a more straightforward way out of the restrictions but also as a way of enhancing public morale. There is uncertainty about the timing of the supply of a safe and effective vaccine and evidence ( Mullard, 2020b) suggests that only 6% successfully come to fruition, which poses a risk for those investing in research and development although there is evidence of hopeful results from phase1/2 trials ( Bar-Zeev & Moss, 2020). The scientific narrative about the time it will take to develop an effective and safe vaccine has varied between 6 and 18 months, although it is reported that a new vaccine takes on average ten years to develop ( Mullard, 2020a).

There is global competition in the race to develop a vaccine and there are currently ten vaccines at the stage of clinical trial although some are being fast tracked with trialling and manufacture being carried out at the same time ( Mullard, 2020b). This raises the question of how far drug companies will be transparent and willing to make public their trial results and share data and results in a coordinated effort. Certainly, given the global nature of the pandemic involving, at least at the present, mainly high-income countries, there may be more of incentive for drug companies to develop and manufacture a vaccine quickly compared with the Ebola epidemic ( Guzman, 2018; Tambo *et al*., 2015), which involved mainly poor resourced low- and middle-income countries. However, given the level of public investment in vaccine development for Covid-19 the expectation is that the vaccine will be universally accessible although this will depend on nationalistic ( Milne & Crow, 2020), geographical and commercial interests such as pricing and profitability ( Mullard, 2020b).

The level of vaccine hesitancy or resistance in some countries ( Wellcome Trust, 2019), suggests participation in a public health vaccination programme may not be attractive to all although this vaccine might be targeted at the adult population rather than children. Recent survey evidence ( You Gov, 2020) shows the majority of people in Britain (71%) say that they would be willing to have a Covid-19 Vaccine with the over-65s being more likely to wish to be vaccinated. However, the priority placed on the treatment and management of Covid-19 patients has led to a reduction in public participation in other vaccination programmes for key diseases such as measles as it has with other health services.

### The influence of the media

The strong emphasis on social distancing and social isolation in the national government policy response to Covid-19 has placed an increasing public reliance on the traditional and social media for sources of information ( Survation, 2020). The role of the media tends to be in framing and setting agendas for newsworthy health policy stories, which can shape both public opinion and more directly government policy, although governments can also use the media, particularly state funded media, to communicate their political messages ( Calnan, 2020b). The media’s portrayal or framing of the government policy response has primarily come through BBC television’s live streaming of daily briefings to the news media and more generally the public. This is one way of ensuring the government remains accountable for its policy under critical scrutiny from the press, or least some sections of the press, particularly when parliament had been in recess due to social distancing restrictions. More recent briefings began to include selected questions from the ‘public’. This is also a means for Government to use the media to get their message across about protecting the NHS, to promote their policy and to be seen to be actively trying to combat the virus (through, for example, the building of new hospitals) a form of symbolic policymaking.

The media briefings particularly used graphs portraying statistical trends in social distancing, infection and deaths, which aim to illustrate the scientific approach being taken and encouraging, at least some members of the public, to become ‘armchair’ epidemiologists ( Rhodes & Lancaster, 2020). The format of the briefing was managed by the government through the public service medium of the BBC, even though the latter had an uneasy relationship with the government prior to the pandemic. However, while some of the questions from the media were critical in relation to the lack of systematic testing or patchy distribution of PPE, or the alleged breaking of the lockdown rules by the government advisor Dominic Cummings, there was little scope for confrontation or pressing if questions were not answered. Different ministers lead the briefing perhaps to reflect that it is not just a public health problem but has wider social, legal and economic implications. The briefings may aid political visibility, transparency and accountability but the variations in the quality of the communication strategy has not enhanced credibility or trustworthiness. Survey evidence shows the broadcast media, especially the BBC, were more trusted then the newspapers ( Survation, 2020), although it has been shown that the public generally adopt a critical or sceptical stance with the media and the importance of trust may be overstated in this context ( Calnan, 2020b). However, more recent evidence shows trust in news organisations is in decline, from 57% to 46% ( Fletcher *et al*., 2020), with the public generally avoiding news broadcasts which has led to the televised press briefings being reduced to weekdays only and more recently to occasions when significant developments are to be announced.

In relation to the social media, there is recognition of its important role for representing diverse voices and increasing the accountability of government ( Limaye *et al*., 2020). Yet this needs to be balanced against concerns ( Open Knowledge Foundation, 2020) about misinformation about inside information about secret plans and health service failures and peddling conspiracy theories, e.g. that 5G is linked to coronavirus. For example, NHS England announced measures in partnership with Google, Twitter, Instagram and Facebook to combat “fake news” about coronavirus. They include Google search pointing people first to verified NHS guidance when looking for “coronavirus treatments” or “coronavirus symptoms”; and working to suspend accounts producing false information ( West, 2020). Misinformation about the risks of vaccines primarily through the social media have fuelled the propaganda of the anti- vaccination movement which may also become prominent if and when a vaccine becomes available for Covid-19 ( Calnan, 2020b).

### The influence of commercial interests

Finally, government policy has not only consequences for the economy as whole but also for social and economic inequalities. The lack of early state intervention might have reflected the neoliberal values of a Conservative government and their reluctance to intervene, and also that they were initially thinking of adopting a mitigation strategy which may have avoided the need for some of the severe social distancing measures that are now having an impact on the country's social and economic life. The five tests set out by the government which they argued needed to be met before relaxing the restrictions focused on health consequences, i.e. on minimising deaths. There was, however, pressure on the government to lift the restrictions owing to concern about the negative impact on the economy (now in recession) which the evidence suggests has experienced considerable, potentially long-standing damage in terms of a loss of productivity. It has been argued that some of these have yet to be met, such as being confident that there will be no second spike and that there are no problems with testing capacity and supplies of personal protective equipment ( O’Dowd, 2020). Thus, it appears that the recent relaxation of the measures were driven by a political decision to meet social and economic interests rather being tied to the science and meeting the criteria outlined in the five tests ( Abbasi, 2020b). Certainly, there is much criticism that the scientific evidence has been ignored in these recent policies and that England is not currently well positioned for a roll back of the lockdown compared with other countries ( Hale *et al*., 2020). However, as the following quotation suggests, there may need to be a trade-off between the public health and the health of the economy although the latter also has consequences for both physical and mental health in the longer term:

*“The government’s decisions about easing the restrictions should be guided not only by a desire to minimise deaths from Covid-19 but also by a desire to minimise all avoidable deaths and to maximise living standards. Exactly how much weight is placed on each of these objectives is a political judgment and will depend not only on the average costs and benefits of interventions that might be incurred or accrue across the population but also on how those are distributed.”* ( Tetlow *et al*., 2020 p16).

Yet, a counter argument is evident in relation to the introduction of the new quarantine laws for visitors to England. Public health concerns appear to have taken priority over the possible economic damage to the travel industry, although this policy has led to criticism and pressure to change these laws particularly from the aviation industry. However, these quarantine rules have recently been revised for visitors to this country from selected countries.

Broader analysis ( Economist, 2020) of the impact of Covid-19 on the economy has highlighted both gains and losses. The crisis is, according to this analysis, set to enhance three trends. First, there will be a quicker adoption of new technologies. Second, global supply chains will be redesigned, accelerating the shift since the trade war began with a critical mass of production close to home using highly automated factories. Finally, there will be a further rise in corporate concentration as government expenditure is taken up by the private sector and large companies grow even more dominant.

The hardest hit, both in the short and longer terms, from the disruption to the economy are the increasing number of people living in precarious social and economic circumstances. Survey evidence showed that not only had 19% lost their jobs but a significant number were or expected to have financial problems ( Duffy, 2020). A report from the Resolution Foundation ( Gustafsson & Mcurdy, 2020) identifies 8.6 million key workers (almost four million health workers, along with education, and food and pharmaceutical retail staff) and 6.3 million people in shutdown sectors whose health and economic position is most at risk. Women are twice as likely to work in these in key worker roles as men (36 vs 18%), including two in five working mothers. Lower earners, those in the bottom half of the earnings distribution, are twice as likely to be key workers, and 2.4 times more likely to work in shutdown sectors, than they are to work in jobs which are likely to be able to be carried out from home.

There is strong evidence of social inequalities in the patterning of disease ( Calnan, 2020b) and Covid-19 is no exception and inequalities have been both replicated and exacerbated. The social inequalities in the risk of serious illness and death from Covid-19 ( ONS, 2020b) might be best explained less in terms of vulnerability to infection, the medicalised explanation, but more in terms of the social resources and living and working circumstances which enable or stand in the way of managing the illness. This might explain the relatively higher death rate amongst BAME ethnic groups reflecting systemic injustice ( Platt & Warwick, 2020; Public Health England, 2020). Analysis of the social class inequalities in relation to Covid-19 ( Arber & Meadows, 2020) suggest a cruel irony in who initially transmitted it and who are most vulnerable:

*“It is a cruel irony that the initial spreaders (or seeds) of the CV-19 pandemic were business people and the affluent (in other words, the middle class), but that the greatest causalities of the pandemic will be the poor and disadvantaged in western countries, and especially the populations of poorer countries.”*

More recent evidence ( ONS, 2020c) shows that the impact of the lockdown on people’s lives varies by income group. Those in high-income households have seen the greatest fall in travel time and a corresponding rise in time spent working from home. They also report having more free time than normal. However, people in low-income households were more likely to continue working outside the home, their increase in free time was smaller than higher-income households and time spent working away from home was unchanged.

## Conclusion

In conclusion the aim of this opinion paper has been to characterise the current health policy response adopted in England to control or manage the Covid-19 epidemic and to identify the key sociological and political influences which have shaped these policies to date. It is based on insights about policy developments as they unfold, which provides a unique opportunity to provide a prospective account although it cannot draw on retrospective data and hindsight which could portray a broader, critical overview of the policy in the future. The overall policy proposed by the government in England to date converges with many other countries in Europe, although it required a shift in emphasis earlier on for this to happen. Its communication strategy has been inconsistent at some points, particularly with regards to exit plans. The policy discourse has been strongly characterised by the management of uncertainty with a renewed trust in scientific and medical advice and expertise, although the link between policy and science has loosened of late. Along with the influence of medical scientific expertise an interplay of other powerful interests has shaped policy including NHS managers, professionals and staff, social care providers, the public, the media, the drug industry and broader commercial interests. However, the reactive nature of this policy needs to be understood within a broader sociohistorical context in which there has been chronic underinvestment in both the NHS and in social care over the last decade ( Calnan, 2020). The state, contrary to the neoliberal values of the current government with its preference for reliance on the market and deregulation, is playing an increasingly interventionist role in the social and economic life of the population through propping up the market and controlling social practices although it is difficult to judge how far these policies will be maintained and if the structure of the economy has been significantly reshaped or will revert back to ‘business as usual’.

The Government has recently committed to an independent inquiry ( Iacobucci, 2020c) into their management of the pandemic so to what extent have these policies been successful? Social distancing and isolation policies have been adhered to by a large majority of the population ( Duffy, 2020), the infection transmission rate has declined, at least in the community, and although the number of deaths from Covid-19 appears to have peaked (the first peak of several?) they are running at one of the highest in the world with social inequalities in the risk of serious illness and death. However, the key evidence to assess success, or relative success compared with other countries, will be the excess mortality data which should be available in the longer term. Preliminary evidence from analysis of excess mortality in Europe ( Voce *et al*., 2020) shows the countries who ‘locked down’ earlier had fewer deaths and that Britain had one of highest death rates per population ( McConalogue & Knox, 2020).

## Data availability

No data are associated with this article.
