# Supplementary material for: Health policy and controlling Covid-19 in England: sociological insights
Source: Emerald Open Res. 2020 Jul 29;2:40. doi: 10.35241/emeraldopenres.13726.2 (PMC7371407; doi:10.35241/emeraldopenres.13726.2)
Supplement: Peer review history

# Peer review: Reviewer response for version 1

**DOI:** 10.21956/emeraldopenres.14801.r26959

**Published:** 2020-07-16

**Authors:** Mike Saks

**Affiliations:** 1 University of Suffolk, Ipswich, UK

This was one of the most informative and cogent articles that has appeared on health policy on Covid-19 in England. Despite its devastating consequences in one of the largest crises worldwide this century, so many articles and books that are being published avoid reference to the pandemic. This piece not only addresses the approach to Covid-19 head-on, but is well-written, clearly set out and covered all the main bases in articulating government policy in England and discussing the various influences on it. The article is also justly critical of aspects of the policy adopted - not least by evaluating it in an international context, in comparison with the stance taken by other governments particularly in Europe.

 Against this, it should be noted that, in describing the multiple facets of the health policy adopted in England regarding Covid-19, there have been a number of significant changes since this publication. These can be illustrated by face masks being required in shops, the construction of travel bridges to selected countries without mandatory quarantine arrangements, and local lockdowns to deal with upsurges in the virus - such as in Leicester related to unhealthy working conditions in sweatshops in the clothing industry. This, though, is not so much a criticism of this paper as an indication of just how fast moving the policy agenda in this field has been.

 Other points include the need in a sociological commentary for greater stress to be given to the use of science and evidence-based medicine as a legitimating ideology in the politics of health. More might also have been said about how government has employed the professions with their ethical codes to engender trust as a smokescreen for the shortcomings of its own policy. Here too the critique of state policy might have been extended with reference to the frequently underused concept of 'responsible leadership', which - alongside the Sustainable Development Goals - is now part of the framework adopted by the United Nations (see www.responsible-leadership.org). A more forensic analysis might also have emphasised the longer term treatment of the social care workforce as second class citizens - as amplified by the restricted entry criteria that are presently being imposed on international staff in this sector which will limit the supply of paid carers at a time of serious shortages.

 Otherwise the paper very much hits the mark in its critical analysis. Amongst other things, it strips bare the idea of cohesive scientific advice underpinning government policy, the slowness of the initial response by the government, the absence of a consistent communication strategy, the lack of learning from other countries, and the poor preparedness in public health terms - highlighted by shortages of personal protective equipment and testing procedures. The paper also accentuates that in terms of future treatments and vaccines we cannot rely on the cooperative benevolence of the pharmaceutical industry. Finally, it has the virtue of accessibility, although as a sociological analysis it was a little disappointing that there was no explicit theoretical wrap around to frame this incisive analysis.

Is the topic of the opinion article discussed accurately in the context of the current literature?

Yes

Are arguments sufficiently supported by evidence from the published literature?

Yes

Are all factual statements correct and adequately supported by citations?

Yes

Are the conclusions drawn balanced and justified on the basis of the presented arguments?

Yes

Reviewer Expertise:

Health, professions, regulation and research methods

I confirm that I have read this submission and believe that I have an appropriate level of expertise to confirm that it is of an acceptable scientific standard.

# Peer review: Reviewer response for version 1

**DOI:** 10.21956/emeraldopenres.14801.r26925

**Published:** 2020-07-09

**Authors:** Nelson Filice de Barros

**Affiliations:** 1 Faculty of Medical Sciences, University of Campinas (Unicamp), Campinas, Brazil

The paper began with a descriptive account characterising the current health policy response adopted in England to control or manage the global Covid-19 pandemic. Drawing on the theoretical approach based on insights about policy developments as they unfold, the article provides opportunity to explore a prospective account which could portray a broader critical overview of the policy in the future. The author considered the influence of the key players that took part in the government policy shaping, like: the government and its representatives, NHS managers, scientific advisers and the scientific community, the medical profession, their representatives and clinical practitioners, the allied professions including nursing practitioners and social care agencies and workers, the private health and social care sector, the commercial sector who produce vaccines, ventilators and personal protective equipment, the media in the public and private sectors and the public, patients and their informal carers.  To the author the government policy response might be described as reactive, convergent with many other countries in Europe, communication strategy muddled, strongly characterised by the management of uncertainty with a renewed trust in scientific and medical advice and influenced by the interplay of powerful interests. Part of this characteristics are pointed by the author as the consequence of a broader sociohistorical context in which there has been chronic underinvestment in both the NHS and in social care over the last decade. Another part shows the state playing an increasingly interventionist role in the social and economic life of the population, contrary to the neoliberal values of the current government. With all this deep reflections the author built an important “strategic assessment” about the health policy response adopted in England to control or manage the global Covid-19 pandemic. However, unfortunately, the author did not develop a “tactical adaptation” or “a minimal but paradigmatic programme (…) thinking in terms of a broad strategic historical alternative perspective” to keep, e.g., the current government investment in the NHS and social care. We know that the union of forces to manage a catastrophe dissipate as soon as the problem is controlled, but, at the end, somethings always keep different and here there is an opportunity to build new policy development or social perspective to intervene at the hegemonic practices. In other words, the challenge to the author and all of us facing the “socio-logic” of this pandemic is to propose, possibly, something like to build a wall, which “includes the things we say, a model in the head, and the things which we do with our bodies” ^1^.

Is the topic of the opinion article discussed accurately in the context of the current literature?

Yes

Are arguments sufficiently supported by evidence from the published literature?

Yes

Are all factual statements correct and adequately supported by citations?

Yes

Are the conclusions drawn balanced and justified on the basis of the presented arguments?

Yes

Reviewer Expertise:

Sociology of Health, Cultural Studies of Health, Complementary and Alternative Medicine, Medical Education

I confirm that I have read this submission and believe that I have an appropriate level of expertise to confirm that it is of an acceptable scientific standard.

## References

[1] : Culture and Power.1997; Reference source
